# Supplementary material for: Seagrass deformation affects fluid instability and tracer exchange in canopy flow
Source: Sci Rep. 2023 Mar 8;13:3910. doi: 10.1038/s41598-023-30401-9 (PMC9995504; doi:10.1038/s41598-023-30401-9)
Supplement: Supplementary file 5 — Supplementary Information 5. [file 41598_2023_30401_MOESM5_ESM.pdf]

Supplementary information A

Seagrass deformation affects fluid instability and tracer exchange in canopy flow (Vieira, Allshouse & Mahadevan, 2022)

Comparison between buoyancy and rigidity models

Prior to the onset of instability, or closer to the inflow section of the channel, the flow and grass are both steady. The steady state solution is a function of z alone, and can be calculated with a simplified one-dimensional coupled model that eliminates dependence in x and t (Methods Section).

The steady state velocity profiles $\overline{u}(z)$ and corresponding grass positions $(\overline{x}_g, \overline{z}_g)$ calculated for $Re = 10^3, r = 0.5, \lambda = 1, Fr^2 = 0.1$, are presented for a range of values of the buoyancy parameter β (Fig. S1). Solutions are computed for two different fluid boundary conditions: $\mathrm{d}\overline{u}/\mathrm{d}z = 0$ (free-slip) at the bottom ($z = 0$) (Fig. S1(b)) and $\overline{u} = 0$ (no-slip) (Fig. S1(c)). At the surface ($z = 1$), $\mathrm{d}\overline{u}/\mathrm{d}z = 0$.

As a comparison, Fig. S1(a) presents the solution of Wong *et al.*[33] for neutrally-buoyant blades with flexural rigidity EI and a free-slip bottom boundary condition, where the parameter controlling the blade deformability is the Cauchy number $C_Y = \rho b C_D H^3 U^2/EI$. In our model, $EI \to 0$, $C_Y \to \infty$, buoyancy is the dominant agent that resists the fluid drag on the blade, and therefore β quantifies to what extent the blade can deform. For these steady-state solutions, β is varied while keeping Re, λ and r constant. Note that β and Re can be adjusted without modifying any of the other dimensionless groups by tuning the blade dimension d and ν^*, respectively.

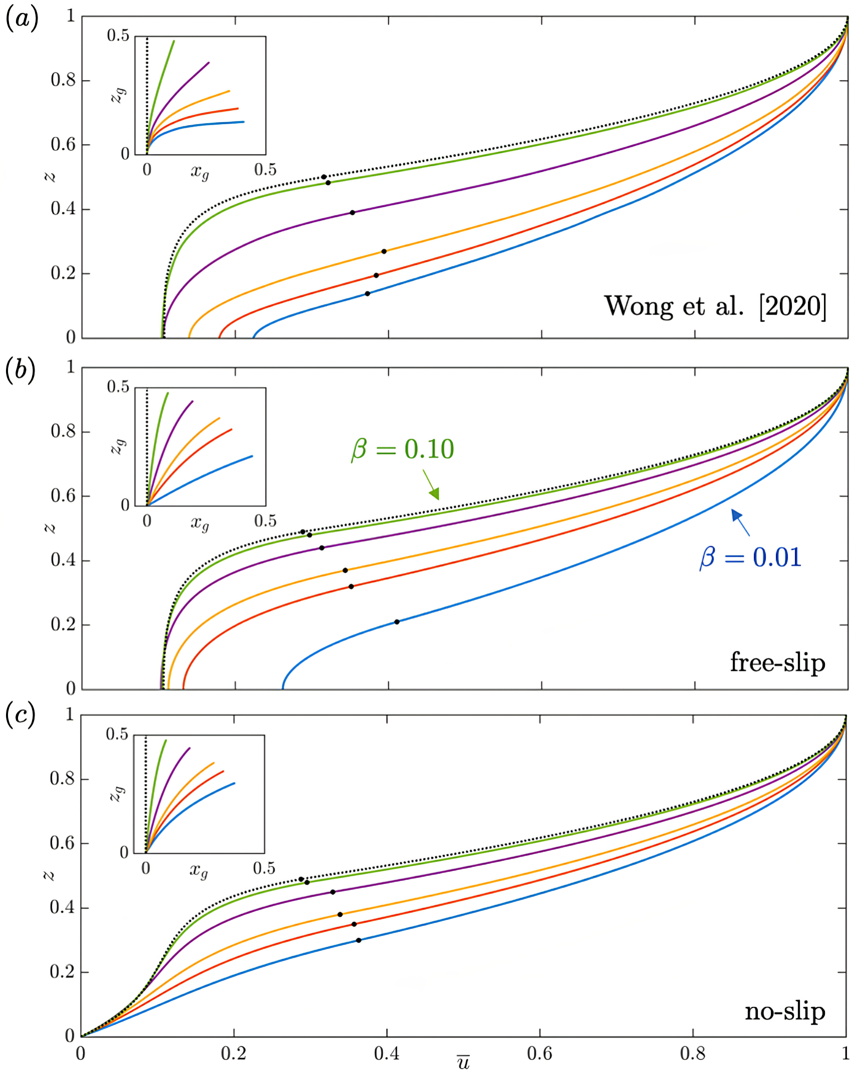

Figure S1. Steady-state horizontal velocity profile $\overline{u}(z)$ and corresponding blade configurations (inset), for $Re = 10^3$, $r = 0.5$, and $\lambda = 1$. (a) Results from Wong *et al.*[33], flexible grass blade, with $C_Y/Re^2 = 10^{-1}$, $10^{-1.5}$, 10^{-2}, $10^{-2.5}$, 10^{-3}, and 0 (dotted). Buoyant grass model, using a (b) free-slip bottom and (c) no-slip bottom boundary condition, and $\beta = 0.01$, 0.015, 0.02, 0.04, 0.10, and ∞ (dotted lines). Same β values are plotted using the same colors in (b,c). The black dots on the velocity profiles mark the position of the corresponding canopy height $\overline{h}_g$ in each case.

Grass blades with bending stiffness (Fig. S1(a)) and buoyancy (Fig. S1(b)) result in qualitatively similar solutions, with the main difference arising from the clamped bottom boundary condition in Wong *et al.*[33] that prevents deflection, compared to the hinged bottom boundary condition we used. The hinged condition results in lower drag near the bottom boundary. Another difference is the fluid velocity at the tip of the grass. For the buoyant model in Fig. S1(b), we observe a monotonic growth of the velocity at the tip with decreasing β, while in Fig. S1(a) there is a peak and then a decay for increasing C_Y. Additionally, small variations of β, as $\beta \to 0$, drastically change the steady-state solutions (note the differences for $\beta = 0.01$ and 0.015 in Fig. S1(b), for example).

The no-slip bottom boundary condition (Fig. S1(c)) reduces blade deflection at the root, resulting in a smaller range of grass deflection angles and ultimately less deflection at the tip. The shape of the velocity profile near the canopy top and the velocity shear magnitude are not sensitive to the bottom boundary condition choice. The no-slip condition at $z = 0$ is used for the time-dependent simulations as it is physically more accurate.

Supplementary information B

Seagrass deformation affects fluid instability and tracer exchange in canopy flow (Vieira, Allshouse & Mahadevan, 2022)

Vortex merger events and tracer exchange

At combinations of high Re and high β, larger-scale time fluctuations in $\Phi(t)$ become more apparent (Fig. 8). To understand what contributes to these additional time scales to the flow, we study the vortex structure and tracer concentration fields for $Re = 1500$. The simulations presented in Fig. S2 only vary the buoyancy parameter, with the left and right panels corresponding to $\beta = 0.06$ and $\beta = 0.20$, respectively. Figs S2(a,b) present the vorticity ζ, and Figs S2(c,d) the tracer concentration C, both at time $t = 300$. These plots highlight how the vorticity field lines up accurately with the tracer field at a given time, even though the tracer concentration has been evolving for $t \in [0,300]$.

While for $\beta = 0.06$ (Figs S2(a,c)) all vortices look similar and periodic, from both vorticity and tracer perspectives, the $\beta = 0.20$ results (Figs S2(b,d)) show signals of vortex interaction, with an imminent vortex merger[37] that has started at $x \approx 26$ (see Supplementary information for a video of the time evolution of these merger events).

Figs S2(e,f) present Hovmöller diagrams of $w(x,z=\overline{h}_g,t)$ for both cases, similar to the ones in Figs 4(a,b). A periodic signal with vortices propagating at constant speed and amplitude is observed for the more deformable blade case (Fig. S2(e)), while more nonlinear interactions and vortex merger events are observed for the less deformable case (Fig. S2(f)). Both cases have a similar dominant vortex speed that is again close to the 0.6 obtained for $\beta = 0.10$ in Fig. 4(b). However, the w amplitudes for $\beta = 0.20$ are stronger, more variable, and the periodicity of the signal is less apparent. This variability may be due to bigger vortices for $\beta = 0.20$ growing enough to interact with neighboring vortices, causing merger events and other nonlinear phenomena that reduce periodicity in the flow. No preferred frequency for vortex merger events was observed.

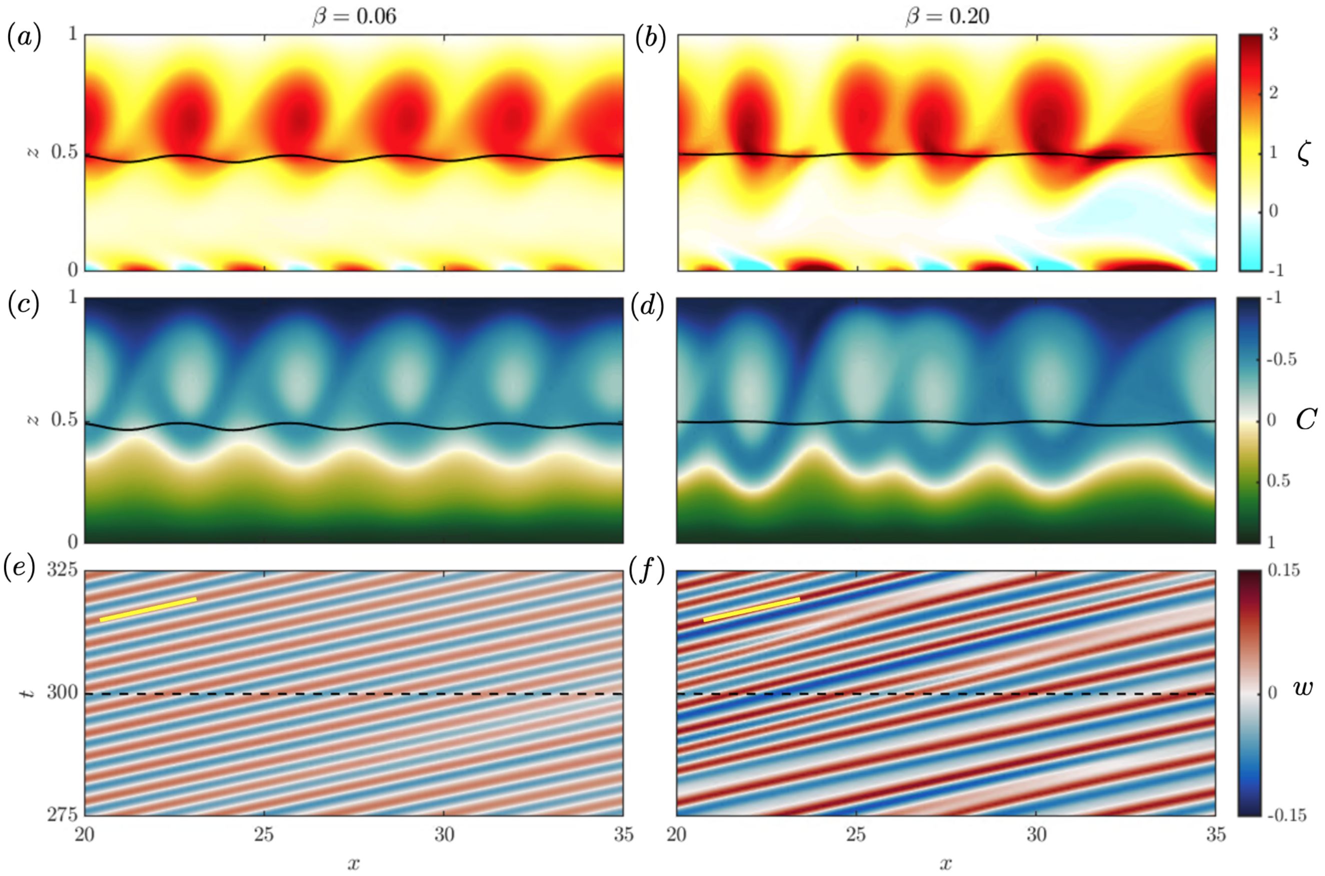

Figure S2. Vortex interaction for variable β and fixed $Re = 1500$. Instantaneous (a,b) vorticity $\zeta(x,z,t=300)$ and (c,d) scalar field $C(x,z,t=300)$. (e,f) Hovmöller diagrams presenting the space and time evolution of $w(x,z=\overline{h}_g,t)$ for $\beta = 0.06$ ((a,c,e), more deformable) and $\beta = 0.20$ ((b,d,f), less deformable), highlighting vortex interactions and merger events for $\beta = 0.20$. The yellow slope indicates the reference $\mathrm{d}x/\mathrm{d}t = 0.6$, and the dashed black line marks the time $t = 300$ when the fields are plotted in $(a-d)$.

Supplementary information C

Comparison to previous experimental work

In this section we present a direct comparison between our simulation results on the relative position between vortices and seagrass bed perturbations (as depicted in Fig. 5) and the experimental observations in Ghisalberti & Nepf[17]. In the experimental investigation, they phase averaged the velocity profile, grass height, and Reynolds stress. Here we reproduce their figures and provide direct comparison to our simulated results.

First, we compare the velocity profiles and the seagrass height. The experimental result is broken into eight phases as the vortices pass a fixed point in space. The maximum height of the grass occurs at phases 1 and 8 while the minimum height occurs at phases 4 and 5. Based on Figure S3a phases 1 and 8 correspond to the slowest average velocity throughout the seagrass layer. This is also demonstrated Figure S3c where the velocity profiles at a fixed height at minimum at phases 1 and 8 when the grass is at its highest.

Numerically, the phase averaged velocity profiles in Figure S3b are similar in a number of ways. The slowest average velocity in the grass also occurs at phases 1 and 8. A couple differences arise potentially due to the simulated grass being a larger fraction of the full domain height. The simulation has greater variability in the velocity near the upper boundary than is observed in the experiments. We also see a closer collapsing of the velocity profile immediately above the grass. The grass height profile is presented in Figure S3d where we again see the minimum grass height at phases 4 and 5 and maximums at phases 1 and 8. In this case, the oscillation of the grass relative to the length of the grass is much smaller than the experiments. In the experimental case the amplitude of oscillation is approximately 10% of the grass height while the simulated oscillation is approximately 2%.

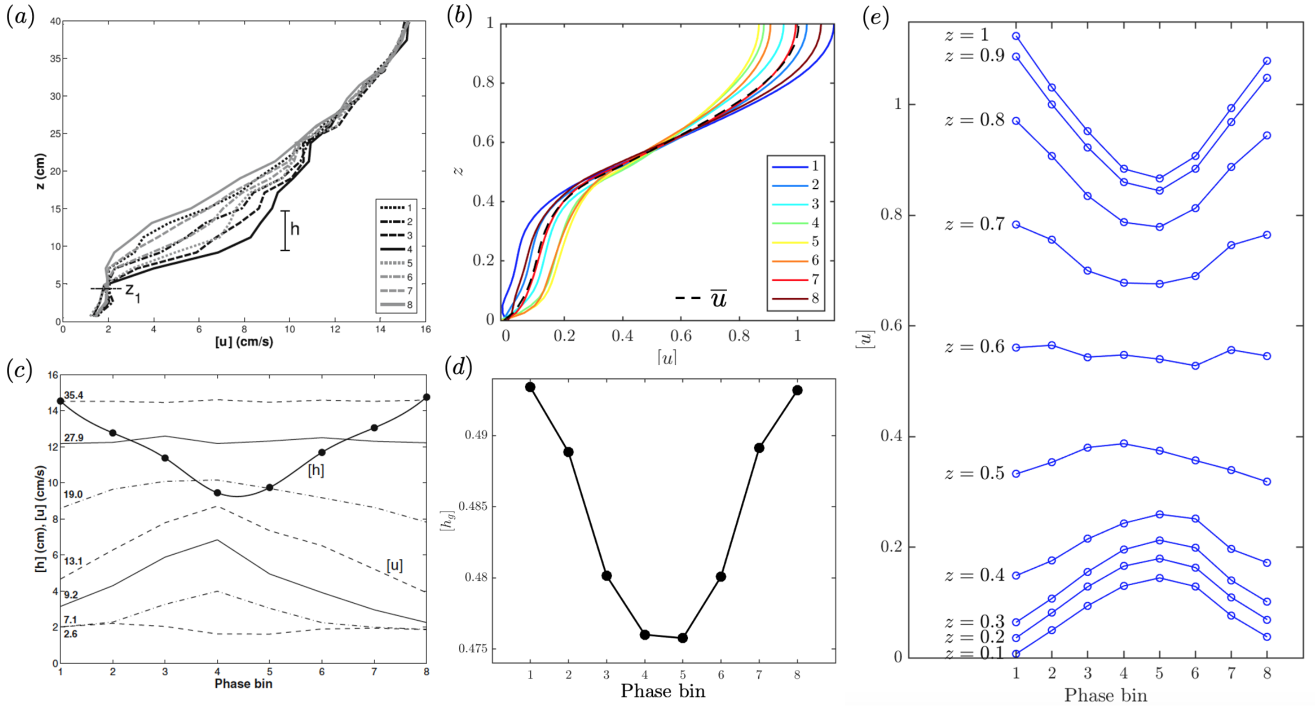

Figure S3. Experimental and numerical comparison of the velocity profile and canopy height at different phases of the vortex passing by a fixed point in space. (a) Reproduction of Figure 7(b) from Ghisalberti & Nepf[17]. presents the phase averaged velocity profile of the water. The blade in this case has a length of 20 cm and has an average height of 12.5 cm. (b) Simulated phase-averaged velocity profiles. Grass height is at the dimensionless $z = .5$ (c) Reproduction of Figure 7(a) from Ghisalberti & Nepf[17] presents the velocity perturbation at fixed heights as well as the grass height at different phases. (d) Simulated grass height. (e) Simulated velocity profiles at a range of fixed heights.

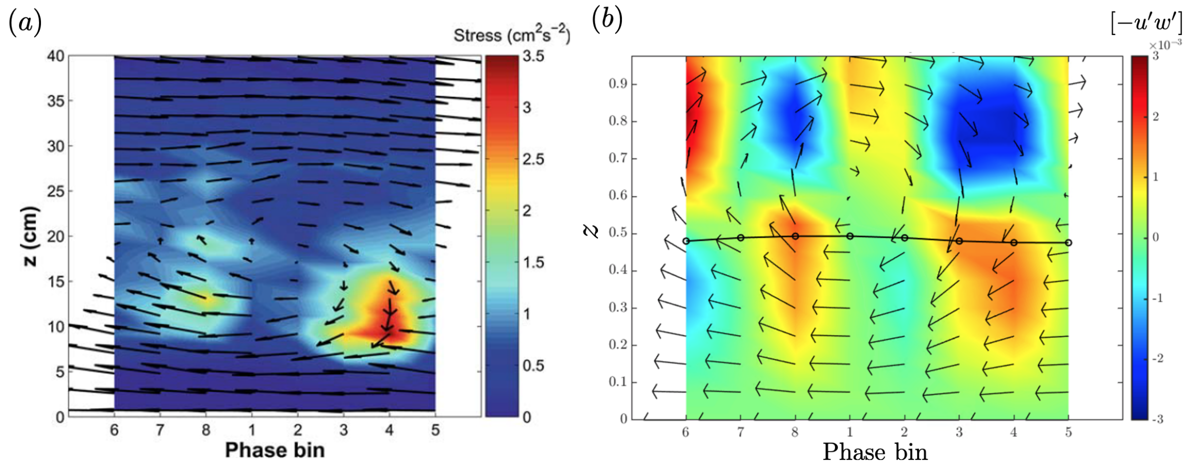

Figure S4. Comparison of experimental and simulated velocity and Reynolds stress fields. (a) Reproduction of Figure 8(a) from Ghisalberti & Nepf[17] presents the velocity perturbation (arrows) and the Reynolds stress at different phases of the oscillation. Note that the phases have been shifted relative to Figure S3. (b) Corresponding simulation result.

The velocity perturbation fields are presented in Figure S4a. Note that this result does appear more like a vortex but has required a shifting of the phase by approximately half a period relative to Figure S3. Again the numerical and experimental results do qualitatively agree for the average velocity perturbation. There are some discrepancies in the Reynolds stress fields but this may again be the result of the grass being a more significant fraction of the water column height.

Supplementary information D (captions embedded in videos)

Video description

Video 1. Instability onset

"Seagrass deformation affects fluid instability and tracer exchange in canopy flow" (Vieira, Allshouse & Mahadevan, 2022)

(top) Vorticity field ζ for $\beta = 0.10$. (bottom) From left to right: steady-state horizontal velocity $\overline{u}$, horizontal velocity perturbation u', vertical velocity w, and grass blade positions (x_g, z_g), for the designated region of the domain (dashed rectangle). The solid black line is the seagrass height h_g. Video corresponds to Figs $3(a,b)$.

Video 2. Tracer transport

"Seagrass deformation affects fluid instability and tracer exchange in canopy flow" (Vieira, Allshouse & Mahadevan, 2022)

(top-left) Tracer concentration C for $\beta = 0.14$, (bottom-left) vertical tracer flux ϕ, and (right) tracer exchange Φ. The solid black line is the seagrass height h_g. Video corresponds to Figs $7(b,d)$ and $8(a)$.

Video 3. Buoyancy and tracer transport

"Seagrass deformation affects fluid instability and tracer exchange in canopy flow" (Vieira, Allshouse & Mahadevan, 2022)

(top) Tracer concentration C, (middle) vertical tracer flux ϕ, and (bottom) tracer exchange Φ. (left) $\beta = 0.06$ and (right) $\beta = 0.14$. The solid black line is the seagrass height h_g. Video corresponds to Figs 7 and $8(a)$.

Video 4. Vortex merger events

"Seagrass deformation affects fluid instability and tracer exchange in canopy flow" (Vieira, Allshouse & Mahadevan, 2022)

For the $Re = 1500$ and $\beta = 0.20$ case, (top) vorticity field ζ and (bottom) tracer concentration C. The solid black line is the seagrass height h_g. Video corresponds to Figs $S2(b,d)$.
